# Hybridization in Canids—A Case Study of Pampas Fox (*Lycalopex gymnocercus*) and Domestic Dog (*Canis lupus familiaris*) Hybrid

**DOI:** 10.3390/ani13152505

**Published:** 2023-08-03

**Authors:** Bruna Elenara Szynwelski, Rafael Kretschmer, Cristina Araujo Matzenbacher, Flávia Ferrari, Marcelo Meller Alievi, Thales Renato Ochotorena de Freitas

**Affiliations:** 1 Laboratório de Citogenética e Evolução, Departamento de Genética, Instituto de Biociências, Universidade Federal do Rio Grande do Sul, Porto Alegre 91509-900, Rio Grande do Sul, Brazil; brunaszynwelski@hotmail.com (B.E.S.); matzenba@gmail.com (C.A.M.); thales.freitas@ufrgs.br (T.R.O.d.F.); 2 Departamento de Ecologia, Zoologia e Genética, Instituto de Biologia, Universidade Federal de Pelotas, Campus Universitário Capão do Leão, Pelotas 96010-900, Rio Grande do Sul, Brazil; 3 Núcleo de Conservação e Reabilitação de Animais Silvestres, Universidade Federal do Rio Grande do Sul, Porto Alegre 90540-000, Rio Grande do Sul, Brazil; flviaelisaferrari@yahoo.com.br (F.F.); marcelo.alievi@ufrgs.br (M.M.A.)

**Keywords:** Canidae, interspecific hybridization, South America

## Abstract

**Simple Summary:**

In South America, the natural habitats of wild canids have undergone significant environmental disruptions, leading to interactions between these animals and domestic dogs (*Canis lupus familiaris*). Previous studies have documented hybridization between wild canids and domestic dogs in North America and Europe. However, there have been no reports of such hybridization in South America until now. In 2021, a female canid from Vacaria in Rio Grande do Sul State, Brazil, was brought to the Center for Conservation and Rehabilitation of Wild Animals—Preserves. This animal exhibited unusual phenotypic characteristics, displaying intermediate traits between domestic dogs and wild canids. Based on these observations, we hypothesized that this animal might result from interspecific hybridization. Therefore, this study aimed to test this hypothesis using genetic and cytogenetic approaches. Our analysis suggests that the canid under investigation is a hybrid between the pampas fox and domestic dog, but future studies are necessary to investigate additional cases of this hybridization in nature. Therefore, the combination of genetic and cytogenetic markers proved valuable in elucidating this case of hybridization. To our knowledge, this represents the first documented case of hybridization between these two species.

**Abstract:**

Hybridization between species with different evolutionary trajectories can be a powerful threat to wildlife conservation. Anthropogenic activities, such as agriculture and livestock, have led to the degradation and loss of natural habitats for wildlife. Consequently, the incidence of interspecific hybridization between wild and domestic species has increased, although cases involving species of different genera are rare. In Vacaria, a Southern city in Brazil, a female canid with a strange phenotype, which had characteristics between the phenotype of the domestic dog (*Canis familiaris*) and that of the pampas fox (*Lycalopex gymnocercus*), was found. Our analysis suggests that the animal is a hybrid between a domestic dog and a pampas fox, but future studies are necessary to investigate additional cases of this hybridization in nature. This finding worries for the conservation of wild canids in South America, especially concerning *Lycalopex* species. Hybridization with the domestic dog may have harmful effects on pampas fox populations due to the potential for introgression and disease transmission by the domestic dog. Therefore, future studies to explore the consequences of hybridization on genetics, ecology, and behavior of wild populations will be essential to improve the conservation of this species.

## 1. Introduction

Interspecific hybridization among animals has increased over the years, such as in mammals [1], birds [2], and fish [3], among others. Hybridization gives rise to new genotypes by combining sets of isolated genes [4]. However, when a new gene pool lacks local adaptation, hybridization may reduce the fitness of hybrid individuals and populations [5,6,7]. Anthropogenic disturbances and habitat loss have increased interspecific hybridization, both due to altered mating patterns that increase the propensity to hybridize and because they can change the landscape to favor hybrids in disturbed areas [8,9,10]. Continuous hybridization events lead to introgression, the permanent introduction of genes from one species to another [11].

The Canidae family is represented by species that arose and diverged in North America about 40 Ma ago [12]. Currently, the Caninae subfamily is the only representative taxon comprising 12 genera and 36 species [13]. In South America, there are six genera of Canidae: *Atelocynus*, *Cerdocyon*, *Chrysocyon*, *Speothos*, *Urocyon*, and *Lycalopex* [13]. Most of the range of South American canids has suffered a massive environmental disturbance [14,15,16], bringing wild canids into contact with domestic dogs, *Canis lupus familiaris* [14,15,16]. This contact increases the transmission of infectious diseases from domestic dogs to wild canids, threatening their survival [17,18,19,20].

In animals, while hybridization among congeneric species is relatively frequent, hybridization between species from different genera is extremely rare since the formation of reproductive barriers increases with the expansion of divergence time [4,21]. Hybridization among species with separate evolutionary trajectories can homogenize genetic pools and defer the speciation process [22]. For instance, several studies showed the occurrence of hybridization between wild canids [23,24,25] and between wild canids and domestic dogs [23,26,27]. Bohling and Waits [23] found evidence of minimal introgression of dogs (*C*. *lupus familiaris*) and gray wolves (*Canis lupus*) into the coyote population in North Carolina, USA. Vila and Wayne [27] observed dog–wolf hybrids in wild European populations and found no significant introgression of dog alleles into the wild wolf. Hinton et al. [25] hypothesized that size disparities between red wolves and coyotes in North America influence positive selective mating and may represent a reproductive barrier between the two species. To our knowledge, there has yet to be a report of hybridization between dogs and wild canids in South America.

**Figure 1:**
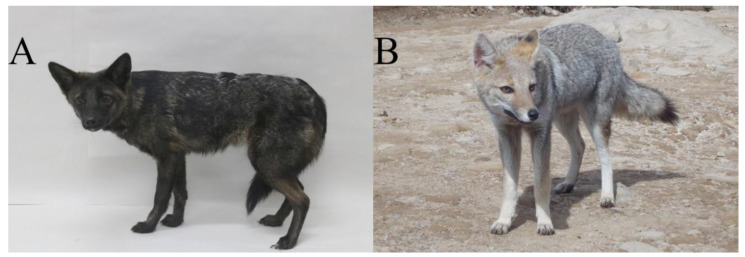
Canid with unusual phenotypic characters investigated herein (**A**) and pampas fox (*Lycalopex gymnocercus*) (**B**). Credits by Thales Renato Ochotorena de Freitas (**A**) and by Bruna Elenara Szynwelski (**B**).

In 2021, a female canid with unusual phenotypic characteristics (Figure 1A), henceforth called ‘canid’, was run over in Vacaria City, Rio Grande do Sul State, Brazil. The canid was transferred to the Center for Conservation and Rehabilitation of Wild Animals (Preservas) of the Veterinary Hospital of the Universidade Federal do Rio Grande do Sul, where it recovered fully. Four species of canids occur in the State of Rio Grande do Sul [28] and display distinct phenotypic characteristics from this animal. The *Speothos venaticus* is a canid with small, rounded ears, short legs and tail, and brown coloring, and its range does not include the region of Vacaria [29,30]. The *Chrysocyon brachyurus* is the biggest canid in South America, weighing about 30 kg and reaching up to 1.6 m. It has long legs and ears, and the pelage of its coat is reddish-brown [31]. These species’ body size and shape were inconsistent with the body shape and size of the canid. Furthermore, only a limited number of occurrence records for *Chrysocyon brachyurus* are known in the State of Rio Grande do Sul [32]. The other two canids in this region are *Cerdocyon thous* and *Lycalopex gymnocercus* [28]. These species have a body shape and size similar to the canid; however, their pelage color is not similar. While the pelage color of *Lycalopex gymnocercus* is yellowish-grey in the coat and light-colored in the legs [33] (Figure 1B), and in *Cerdocyon thous*, it is light gray with a dark stripe in the coat and dark hairs in the paws [34], the pelage color of the canid was completely dark with scarce white hairs.

Because the canid has intermediate phenotypic characteristics between the domestic dog and other wild canids found in Brazil, we hypothesized that it would be a case of interspecific hybridization. To investigate this further, we tested all possible hybridization scenarios involving canid species with distribution in the local area where the canid has been found (Table 1). Thus, our study aimed to apply genetic and cytogenetics approaches to test this hypothesis. Our data suggest that the canid investigated is a hybrid between a pampas fox and a domestic dog. This is the first case of hybridization between a pampas fox and a domestic dog.

## 2. Materials and Methods

### 2.1. Sampling

To obtain skin biopsies and blood to perform cytogenetics and genetics analysis, respectively, the canid (Figure 1A) was sedated with tiletamine and zolazepam (5 mg/kg or 1 mL). A small skin biopsy was collected using a surgical punch (5 mm). The surgical wound was cleaned daily with saline solution, and the stitch was removed after ten days. In the same procedure, 2 mL of blood was collected using a hypodermic needle in the cephalic vein of a thoracic limb.

### 2.2. Cell Culture, Chromosome Preparation, and Cytogenetics Analysis

Fibroblast cell cultures from a skin biopsy were established to obtain metaphase chromosomes following Verma and Babu [36]. Briefly, the cells were grown at 37 °C in Dulbecco’s Modified Eagle’s Medium-high glucose (Gibco), enriched with 15% fetal bovine serum (GIBCO), penicillin (100 units/mL), and streptomycin (100 mg/mL). Chromosome preparations were obtained following standard procedures: 1 h in colchicine, 15 min in hypotonic solution (0.075 M KCl), and fixation in 3:1 methanol:glacial acetic acid. The cell suspension was dropped onto clean slides and stained with Giemsa, 10% in 0.07 M phosphate buffer at pH 6.8 for 5 min, followed by air drying. The diploid number and chromosome morphology were identified by analyzing 50 metaphase chromosomes. According to Sumner [37], the chromosomal regions rich in heterochromatin were identified via C-banding. All cytogenetic observations were made with a ZEISS Axiophot Epifluorescence Microscope (Zeiss, Oberkochen, Germany) and ZEN 2 (Blue edition) software.

### 2.3. Molecular Analysis

DNA was extracted from blood following the phenol–chloroform protocol [38]. The mitochondrial gene COI (551 bp) [39] was amplified to access the maternal inheritance of the canid. Biparental inheritance was accessed using amplifying five nuclear segments, APOB (889 bp) [40], BDNF (542 bp) [41], CHRNA1 (339 bp) [42], GHR (749 bp), and FES (407 bp) [43], previously successfully amplified for South America canids [44]. PCR mix was performed with 1 µL of DNA (50 ng/µL), 0.2 µL of 10 mM forward and reverse primers, 0.2 µL of 10 mM deoxynucleotide triphosphates, 2.0 µL of 10X PCR buffer, 1.5 µL of 50 mM MgCl_2_ polymerase cofactor, and 0.2 µL of 5 U/µL DNA Taq polymerase (Ludwig Biotec, Alvorada, Brazil), for 20 µL in total with the addition of ultrapure water. PCR cycling followed Eizirik [45] for nuclear sequences and Folmer et al. [39] for COI. PCR products were checked in 1% agarose gel. Purification and sequencing in both directions on an ABI 3730 automatic sequencer were performed in Macrogen Inc., Seoul, Korea. The sequences were submitted to GenBank under the accession numbers shown in Supplementary Table S1.

The Basic Local Alignment Search Tool (BLAST) was run for COI and showed the highest similarity with the pampas fox. Due to mitochondrial DNA indicating that the maternal lineage was from pampas fox, we obtained two representative DNA samples of this species from the scientific collection at PUCRS to conduct nuclear genome comparisons. Moreover, representative tissue from two carcasses of domestic dogs was obtained in the Veterinary Hospital of Universidade Federal do Rio Grande do Sul, and the DNA was extracted using the CTAB protocol [46]. To perform mtDNA analysis, we compiled sequences from GenBank for two possible pampas fox parental lineages (access codes MK321457.1, KF572951.1, MK321456.1, MK321455.1, MK321448.1, MK321447.1, MK321442.1, MK321433.1, and MK321411.1) and domestic dogs (access codes MN542345.1, MN542344.1, MN542343.1, MN542342.1, MN542341.1, MN542340.1, MN542339.1, MN542338.1, and MN542337.1).

Chromatograms were inspected by eye, and sequences were edited in Chromas 2.6.6. The alignment used the Clustal W algorithm [47] implemented in Mega X [48]. The haplotype relationships of mtDNA were examined using the Median Joining method [49] in PopART v1.7 [50] using epsilon = 0.

The heterozygous sites of nuclear segments were identified in the chromatograms, and they were represented using the ambiguity codes following the International Union of Pure and Applied Chemistry. A BLAST was run for each nuclear segment of the canid, and the sites in which the dog and pampas fox presented exclusive polymorphisms and were homozygous for them were represented in a table with the polymorphisms found in the canid.

### 2.4. Photographs Analysis

We conducted a thorough search on INaturalist to investigate whether any individuals with similar characteristics to the canid (Figure 1A) have been previously reported in *L. gymnocercus* (Figure 1B) photographs.

## 3. Results

### 3.1. Chromosomal Analysis

**Figure 2:**
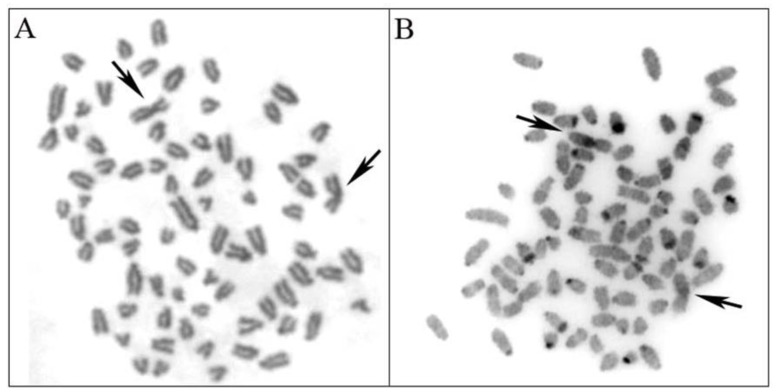
Giemsa-stained (**A**) and C-banded (**B**) metaphase in a hybrid canid showing 76 chromosomes. The chromosome number is intermediate between that of the domestic dog (*Canis lupus familiaris*, 2n = 78) and of the pampas fox (*Lycalopex gymnocercus*, 2n = 74). C-band positive heterochromatic appear as dark signals (**B**). Arrows signal the two X chromosomes (XX).

The female individual analyzed showed a karyotype with 76 chromosomes (Figure 2A), with the autosomes acrocentric and one X chromosome submetacentric and the other X chromosome metacentric. Heterochromatin was found at the centromeric regions of 13 autosomes, at the telomeric regions of 12 autosomes, and at the interstitial regions of another two autosomes (Figure 2B). The submetacentric X chromosomes showed a conspicuous block of heterochromatin subcentromeric on its long arm, while the metacentric X chromosome displayed a weak signal at the same region in all metaphases analyzed (Figure 2B).

### 3.2. MtDNA Analysis

**Figure 3:**
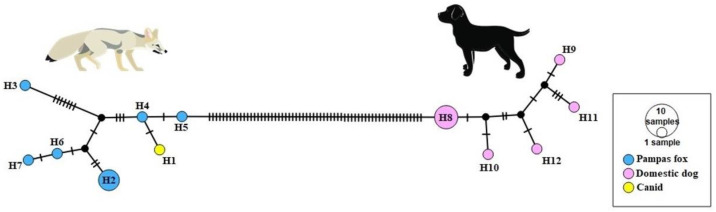
Haplotype relationships for mtDNA COI among pampas fox (*Lycalopex gymnocercus*), domestic dog (*Canis lupus familiaris*), and the canid.

Maternal inheritance, represented by mitochondrial lineage, shows that the canid has pampas fox maternal ancestry. In total, we obtained 551 bp fragments of the mitochondrial gene COI, where 550 sites were identical to the pampas fox reference used MK321448.1 haplotype (H4). The exception was at one site (23rd position), where the canid haplotype has a polymorphism that had not been previously observed in the domestic dog and pampas fox haplotypes. Nevertheless, the canid was clustered with all pampas fox sequences in the haplotype network. Domestic dog sequences were isolated from the pampas fox/canid cluster by 62 mutational steps (Figure 3).

### 3.3. Nuclear Analysis

Among five nuclear segments used to investigate biparental inheritance, four showed polymorphisms. The BDNF segment was not polymorphic and was excluded from our results. The polymorphic sites of the four polymorphic genes are represented in Table 2.

The polymorphic sites should be distinct and homozygous between the pampas fox and dog to support our hypothesis. That way, if the canid is a hybrid between these species, it should have a heterozygous site reporting the base found in the pampas fox and the base found in the dog. A total of 13 polymorphisms were presented in our nuclear dataset, and the GHR segment had the highest polymorphism (5), followed by CHRNA1 (4), FES (3), and APOB (1). However, the sites 192 and 267 of CHRNA1 were distinct between the two dogs. At site 287 of CHRNA1, the canid had a heterozygous site, while the domestic dog and pampas fox had a homozygous site, all displaying the Cytosine base. At site 110, the canid was homozygous for Thymine, shared by dog and pampas fox. The polymorphic sites of CHRNA1 did not contribute to understanding the biparental inheritance of the canid because they are not distinct and homozygous between the domestic dog and the pampas fox. This also happened at sites 569, 638, 671 of GHR and 308/318 of FES. Biparental inheritance is clear at sites 99 and 446 of GHR, sites 88 and 231/241 of FES, and sites 161 of APOB. In these positions, dogs and pampas foxes were homozygous and had exclusive polymorphisms. Consequently, the canid shows a heterozygous site, reporting both bases from domestic dogs and pampas foxes.

In addition to the single nucleotide polymorphisms described here, an indel (insertion or deletion) was observed in the FES segment. In this segment, the dog had 10 base pairs from site 168 to site 177, which were absent in the pampas fox. Since the DNA strands overlap in the sequencing chromatogram, this 10-base segment derived from the dog appeared to overlap on the pampas fox strand in canid (Figure S1). Including 47 GenBank sequences belonging to 27 canid species in the FES alignment, we observed that the 10-base segment occurs in 25 species of canids (Table S2). The only species that did not show this segment were *Lycalopex fulvipes,* and the sister species of pampas fox, *Lycalopex griseus* (Table S2) [51]. Neither sequence belonging to pampas fox was available in the GenBank database for this segment. Three of these 27 species that presented the 10-base segment occur in the Vacaria City region. They are *Chrysocyon brachyurus, Cerdocyon thous,* and the dog *(Canis lupus familiaris*), but according to cytogenetic data, a hybridization involving *C. brachyurus* or *C. thous* could not result in the karyotype found in the canid (see Section 4).

### 3.4. Photographs Analysis

We discovered a total of 1112 photographs on INaturalist that were registered as *L. gymnocercus*. However, upon reviewing these photographs, we did not come across any specimens with a pelage color resembling that of the canid found in Vacaria City.

## 4. Discussion

This study investigated an intriguing canid individual exhibiting unusual phenotypic characteristics, which could not be associated with any known canid species with distribution in Brazil, according to our photographs analysis. Using genetic and cytogenetic markers, our findings suggest that this individual represents a first-generation hybrid between a dog (*Canis lupus familiaris*) and a pampas fox (*Lycalopex gymnocercus*). This discovery implies that, although these species diverged about 6.7 million years ago [52] and belong to different genera, they might still produce viable hybrids. Hence, these species are isolated by postzygotic barriers, although further investigations are required to determine the fertility of these hybrids.

Cytogenetic techniques offer an easy and cost-effective approach to identifying hybrid individuals [53,54], particularly when the parental species possess distinct karyotypes, as observed in the Canidae family [34,35,55,56,57]. Thus, the initial phase of our study involved determining the number of chromosomes and morphologies of the individual under investigation. We identified 76 autosomal chromosomes, all acrocentric, and the X chromosomes displaying submetacentric and metacentric morphologies. *Chrysocyon brachyurus* is the only canid with distribution in Brazil with 2n = 76 [34]. However, this species significantly differs in physical appearance from the individual under study. The karyotype of the individual closely resembled that of the dog [35] and the pampas fox [34], as both species exhibited acrocentric morphology for their autosomes and a metacentric or submetacentric X, respectively. Nonetheless, dogs possess 2n = 78 [35], while pampas foxes have 2n = 74 [34]. Thus, this finding provided the initial evidence indicating that the individual in question is a hybrid between these species, as the haploid number for dogs (39 chromosomes) and pampas foxes (37 chromosomes) explain the diploid number found in the individual (2n = 76). Additional evidence supporting the hybridization hypothesis was the observation that the canid individual possessed one X chromosome with a submetacentric morphology and the other X chromosome with a metacentric morphology.

We further explored C-banding in the canid. Our analysis revealed that heterochromatin was predominantly located in the telomeric and centromeric regions of several autosomal pairs as well as the X chromosome. The dog is known to have heterochromatin only on the centromeric regions of six pairs [35,58]. On the other hand, the pampas fox exhibits heterochromatin in both the centromeric and telomeric regions of most autosomes [34]. Further, the submetacentric X chromosomes in the canid showed a conspicuous block of heterochromatin in the pericentromeric region, while the metacentric X chromosome exhibited a weak signal in all metaphases analyzed. The submetacentric X chromosome, with a conspicuous block of heterochromatin in the pericentromeric region, resembles the X in pampas foxes [34]. On the other hand, the metacentric X chromosome with a weak signal of heterochromatin resembles the dog X chromosome. Fujinaga et al. [35] did not find the block of heterochromatin, while Manolache et al. [58] found a minor heterochromatic area on this chromosome in dogs. Hence, the C-banding results corroborate the hybridization hypothesis between dogs and pampas foxes.

The analysis of mtDNA revealed that the maternal lineage of the canid belonged to the pampas fox. Additionally, five APOB, FES, and GHR segment polymorphisms were consistent with our cytogenetic and mtDNA data. These segments are specific to each species, so the parental species (dog and pampas fox) exhibited homozygote sites, while the canid showed a heterozygote site with both dog and pampas fox polymorphisms. Although the observed indel in the FES segment is also found in two canids, *C*. *brachyurus* and *C*. *thous*, with a geographic distribution in Vacaria City, the cytogenetic data do not support the hypothesis of hybridization involving these species (Table 1). Moreover, the hybridization between *Canis lupus familiaris* (2n = 78) and *C*. *thous* (2n = 74) would produce a hybrid with 76 chromosomes, but most of the autosomal chromosomes in *C*. *thous* are bi-armed, which is not characteristic of the Canid karyotype, effectively discarding this possibility.

Although our nuclear analysis was limited to only two specimens of the putative parental species, it does not undermine our evidence of hybridization. While it does not offer sufficient information to definitively determine whether the observed genotypes in the canid are the result of hybridization or intraspecific variation within the pampas fox population, it does provide valuable insights into the hybridization event. When we consider our integrative analysis, which includes photographs, cytogenetic data, mtDNA analysis, and nuclear analysis, the possibility of hybridization between the pampas fox and the dog should not be disregarded.

The Pampas biome represents a large proportion of the pampas fox distribution range. However, this species is also common in open woodlands and in modified habitats, such as grazed pastures and croplands [59]. The geographic region where the hybrid was found belongs to the Atlantic Forest biome, the most anthropic biome in Brazil [60,61]. The anthropization of the pampas fox habitat has caused this species to be tolerant of human disturbance [16], increasing overlapping ranges of this species with the domestic dog and may have facilitated the interspecific hybridization between these two taxa.

This study suggests a unique case of hybridization between the pampas fox and the domestic species *Canis lupus familiaris*. Further studies are necessary to examine the frequency of hybridization and the potential for genetic introgression of dog genes into Pampas fox populations once the occurrence of introgression of dog alleles to pampas fox populations means introgression of alleles shaped by artificial selection on species vulnerable to natural selection. The pampas fox has a coat color very similar to its habitat, as shown in Figure 1B, while the hybrid has a very dark coat color (Figure 1A), contrasting with the typical color of the pampas fox. Hybridization and introgression can have harmful impacts on the fitness of wild populations via disrupting local adaptation [8,62]. Further, the possibility of cross-species transmission of canine diseases [63,64] may represent another risk for pampas fox populations, especially since the pampas fox is susceptible to coronavirus, parvovirus, distemper, and brucellosis, diseases associated with the anthropic environments where dogs live [14,64].

## 5. Conclusions

In conclusion, here we suggest the first report of hybridization between a domestic dog and a pampas fox. Genetic management of interspecific hybrids is important to species conservation. This step requires the application of methodologies able to provide an easy and undoubted genetic characterization of parents and hybrids [3]. In this context, the combination of cytogenetics, mtDNA, and the nuclear markers APOB, FES, and GHR was useful to clarify this case of hybridization. Nevertheless, additional efforts are required to investigate hybridization frequency, the possibility of genetic introgression of dog genes into pampas fox populations, and the impacts of this event on genetic, behavioral, and ecological factors of pampas fox populations.

## Figures and Tables

**Table 1:** Hybridization hypothesis tested in our study.

Hybridization Hypothesis	Expected Number of Chromosomes	Cytogenetics Reference
*C*. *lupus familiaris* (2n = 78) X *L*. *gymnocercus* (2n = 74)	76	[34,35]
*C*. *lupus familiaris* (2n = 78) X *C. thous* (2n = 74)	76	[34,35]
*C. lupus familiaris* (2n = 78) X *C. brachyurus* (2n = 76)	77	[34,35]
*C. brachyurus* (2n = 76) X *L*. *gymnocercus* (2n = 74)	75	[34]
*C. brachyurus* (2n = 76) X *C. thous* (2n = 74)	75	[34]
*C. thous* (2n = 74) X *L*. *gymnocercus* (2n = 74)	74	[34]

**Table 2:** Polymorphic sites in nuclear segments. * The polymorphisms in sites 231 and 308 are represented in sites 241 and 318 in the domestic dog, once domestic dogs present a segment of 10 bp after site 167 (see below).

		Polymorphic Sites				
		161				
**APOB**	Pampas fox 1	*				
	Pampas fox 2	G				
	Canid	*R*				
	Domestic dog 1	A				
	Domestic dog 2	A				
		110	192	267	287	
**CHRNA1**	Pampas fox 1	T	C	G	C	
	Pampas fox 2	T	C	G	C	
	Canid	T	C	G	*Y*	
	Domestic dog 1	*K*	*Y*	G	C	
	Domestic dog 2	*K*	T	A	C	
		88	231 *	308 *		
**FES**	Pampas fox 1	A	G	*S*		
	Pampas fox 2	A	G	*S*		
	Canid	*M*	*K*	G		
	Domestic dog 1	C	T	G		
	Domestic dog 2	C	T	G		
		99	446	569	638	671
**GHR**	Pampas fox 1	T	T	*W*	*Y*	C
	Pampas fox 2	T	T	*W*	*Y*	C
	Canid	*Y*	*W*	T	T	C
	Domestic dog 1	C	A	*W*	C	*Y*
	Domestic dog 2	C	A	T	C	C

Heterozygous sites in italics were classified following the International Union of Pure and Applied Chemistry code A or G = R, C or T = Y, G or C = S, A or T = W, G or T = K, A or C = M. * segment not amplified in individual pampas fox 1.

## Data Availability

Not applicable.

## Supplementary Materials

The following supporting information can be downloaded at: https://www.mdpi.com/article/10.3390/ani13152505/s1. Figure S1: Sequencing of the FES segment in the pampas fox (*Lycalopex gymnocercus*), the dog (*Canis lupus familiaris*), and the canid; Table S1: GenBank sequences belonging to species of canids used in the alignment of FES to compare the presence or absence of the 10 bp segment. Table S2: GenBank sequences belonging to species of canids used in the alignment of FES to compare the presence or absence of the 10 bp segment.
