# Supplementary material for: Hybridization in Canids—A Case Study of Pampas Fox (Lycalopex gymnocercus) and Domestic Dog (Canis lupus familiaris) Hybrid
Source: Animals (Basel). 2023 Aug 3;13(15):2505. doi: 10.3390/ani13152505 (PMC10417603; doi:10.3390/ani13152505)
Supplement: Supplementary file 1 [file animals-13-02505-s001.zip › animals-2448242-supplementary.pdf]

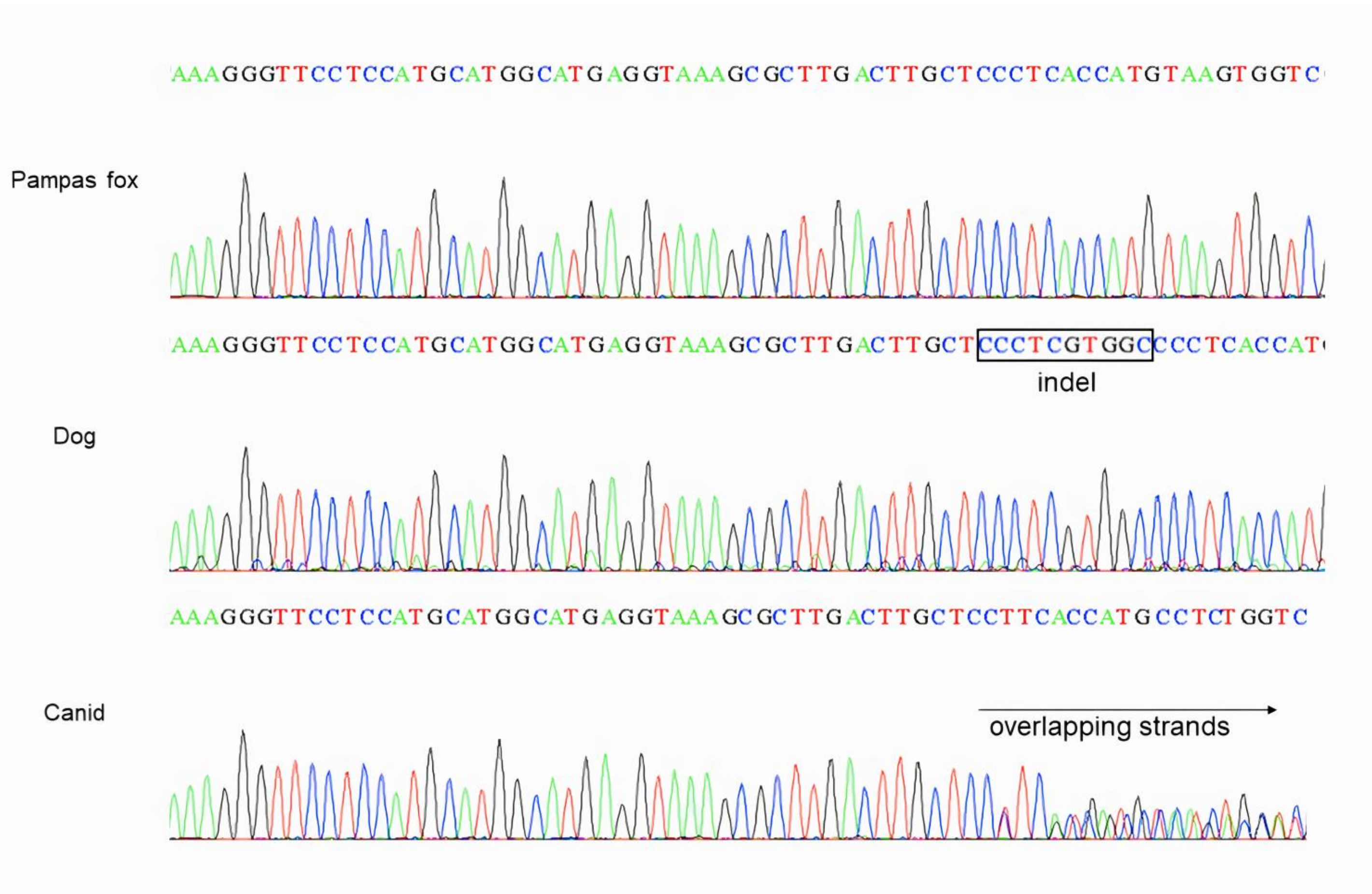

Figure S1. Sequencing of the FES segment in the pampas fox (*Lycalopex gymnocercus*), the dog (*Canis lupus familiaris*), and the canid.

Table S1: GenBank accession codes of sequences amplified in this study.

	APOB	BDNF	CHRNA1	FES	GHR	COI
Domestic dog 1	OR268628	OR294934	OR294939	OR287503	OR282475	
Domestic dog 2	OR268629	OR294935	OR294490	OR287504	OR282476	
Pampas fox 1	-	OR294936	OR294941	OR287505	OR282477	
Pampas fox 2	OR268630	OR294937	OR294942	OR287506	OR282478	
Canid	OR268631	OR294938	OR294943	OR287507	OR282479	OR283802

Table S2: GenBank sequences belonging to species of canids used in the alignment of FES to compare the presence or absence of the 10 bp segment.

	Species	GenBank accession code	Presence of 10 bp
1	*Canis lupus familiaris*	GU931096	Presence of 10 bp
		AY885362	Presence of 10 bp
		EU871598	Presence of 10 bp
		DQ205795	Presence of 10 bp
2	*Canis simensis*	DQ239455	Presence of 10 bp
3	*Canis adustus*	AY885357	Presence of 10 bp
4	*Canis mesomelas*	AY885363	Presence of 10 bp
5	*Canis aureus*	AY885359	Presence of 10 bp
6	*Canis latrans*	AY885361	Presence of 10 bp
7	*Cerdocyon thous*	EF107040	Presence of 10 bp
		EF107041	Presence of 10 bp
		EF107038	Presence of 10 bp
		EF107039	Presence of 10 bp
8	*Lycaon pictus*	AY885370	Presence of 10 bp
9	*Speothos venaticus*	AY885373	Presence of 10 bp
10	*Alopex lagopus*	DQ205796	Presence of 10 bp
		AY885355	Presence of 10 bp
11	*Otocyon megalotis*	AY885372	Presence of 10 bp
12	*Chrysocyon brachyurus*	AY885360	Presence of 10 bp
13	*Atelocynus microtis*	AY885356	Presence of 10 bp
14	*Vulpes zerda*	AY885369	Presence of 10 bp
15	*Vulpes rueppellii*	DQ239462	Presence of 10 bp
16	*Vulpes cana*	DQ239460	Presence of 10 bp
17	*Vulpes macrotis*	AY885376	Presence of 10 bp
18	*Vulpes corsac*	AY885375	Presence of 10 bp
19	*Nyctereutes procyonoides*	GU931097	Presence of 10 bp
		AY885371	Presence of 10 bp
20	*Cuon alpinus*	AY885358	Presence of 10 bp
21	*Urocyon littoralis*	DQ239459	Presence of 10 bp
22	*Urocyon cinereoargenteus*	GU931098	Presence of 10 bp
		AY885374	Presence of 10 bp
23	*Lycalopex vetulus*	DQ239458	Presence of 10 bp
24	*Lycalopex shecurae*	AY885368	Presence of 10 bp
25	*Lycalopex culpaeus*	DQ239456	Absence of 10 bp
		OM169113	Presence of 10 bp
26	*Lycalopex fulvipes*	DQ239457	Absence of 10 bp
27	*Lycalopex griseus*	AY885366	Absence of 10 bp
		OM169104	Absence of 10 bp
		OM169109	Absence of 10 bp

		OM169106	Absence of 10 bp
		OM169111	Absence of 10 bp
		OM169107	Absence of 10 bp
		OM169112	Absence of 10 bp
		OM169108	Absence of 10 bp
		OM169110	Absence of 10 bp
		OM169103	Absence of 10 bp
		OM169105	Absence of 10 bp
